# Outpatient administration of naxitamab in combination with granulocyte‐macrophage colony‐stimulating factor in patients with refractory and/or relapsed high‐risk neuroblastoma: Management of adverse events

**DOI:** 10.1002/cnr2.1627

**Published:** 2022-05-17

**Authors:** Jaume Mora, Godfrey C. Chan, Daniel A. Morgenstern, Karsten Nysom, Melissa K. Bear, Karen Tornøe, Brian H. Kushner

**Affiliations:** ^1^ Pediatric Cancer Center Barcelona Hospital Sant Joan de Déu Barcelona Spain; ^2^ Queen Mary Hospital University of Hong Kong Pokfulam Hong Kong; ^3^ Solid Tumor Program Hospital for Sick Children Toronto Canada; ^4^ Department of Pediatrics and Adolescent Medicine Rigshospitalet Copenhagen Denmark; ^5^ Pediatric Hematology and Oncology Riley Hospital for Children Indianapolis Indiana USA; ^6^ Medical Director Ymabs Therapeutics Hørsholm Denmark; ^7^ Department of Pediatrics Memorial Sloan Kettering Cancer Center New York New York USA

**Keywords:** adverse event, immunotherapy, neuroblastoma, pediatric oncology

## Abstract

**Background:**

Naxitamab is a humanized GD2‐binding monoclonal antibody that received accelerated approval from the U.S. Food and Drug Administration for refractory or relapsed high‐risk neuroblastoma limited to bone or bone marrow. Trial 201 (NCT03363373) is an ongoing global clinical trial to evaluate the efficacy and safety of naxitamab in combination with granulocyte‐macrophage colony‐stimulating factor in this population.

**Aims:**

Here, we review the safety profile and adverse event (AE) management associated with naxitamab administration in a pediatric population, based on Trial 201 protocol guidelines and clinical trial experience.

**Methods and Results:**

At least 50% of patients experienced pain, hypotension, bronchospasm, cough, vomiting, diarrhea, nausea, and tachycardia, with the following reported at grade ≥3 AEs for at least 10% of patients: pain, hypotension, urticaria, and bronchospasm. These AEs were generally manageable in the outpatient setting using premedications, supportive therapies, and appropriate monitoring post‐infusion. Algorithms were established for infusion‐related AEs, including hypotension and bronchospasm, to provide guidance to investigators for early recognition and timely intervention, including medication and infusion rate modification or interruption, or treatment discontinuation, based on AE severity. Educating patients and caregivers on what to expect regarding premedication at home, experience during the infusion cycle, and post‐infusion monitoring helps optimize naxitamab treatment and supportive therapies and may reduce treatment burden.

**Conclusion:**

This article highlights the protocol‐based recommendations for the management of acute AEs associated with outpatient naxitamab treatment in Trial 201. The authors recommend close monitoring and timely implementation of measures to ensure that patients can remain on treatment and obtain maximum clinical benefit from naxitamab therapy.

## 1 INTRODUCTION

Neuroblastoma represents an estimated 10% of all pediatric cancers, accounting for 15% of cancer deaths in children.^1^ Approximately 50% of newly diagnosed patients have high‐risk (HR) neuroblastoma, most commonly due to the presence of metastatic disease involving bone or bone marrow (BM).^1^, ^2^ Despite advances in therapy, long‐term outcomes, particularly for patients with refractory and/or relapsed (R/R) disease, are discouraging and additional treatment options are needed.^3^, ^4^

Naxitamab (previously called humanized 3f8 or hu3F8), a humanized GD2‐binding monoclonal antibody (mAb),^5^ is approved in the USA for use in combination with granulocyte‐macrophage colony‐stimulating factor (GM‐CSF) for the treatment of patients with R/R HR neuroblastoma limited to bone and/or BM who have demonstrated a partial response, minor response, or stable disease to prior therapy. The accelerated approval was based on the interim data from the global clinical trial Trial 201 (NCT03363373) and the phase 1/2 clinical trial Trial 12‐230 (NCT01757626).^6^, ^7^ In both clinical trials, naxitamab was administered in the outpatient setting (defined as without the need for overnight hospital stay for infusion). In Trial 201, this accounted for 96% of all infusions.^8^, ^9^

Naxitamab infusion duration, schedule, and setting are distinct from other approved anti‐GD2 mAbs, such as dinutuximab and dinutuximab beta. Naxitamab is administered as a 30–60‐minute infusion on Days 1, 3, and 5 of each cycle, whereas dinutuximab involves longer infusions (10–20 h/day for 4 consecutive days, requiring inpatient administration).^10^ Dinutuximab beta can be administered either as an 8‐hour infusion for five consecutive days in an inpatient setting, or as a continuous infusion over 10 days, potentially facilitating at‐home treatment via a portable infusion pump.^11^ The infusion time for naxitamab is based on dose–response data from preclinical studies that demonstrated a shorter duration resulted in higher serum antibody levels that correlated with better efficacy against tumor cells, supported by early studies looking at mu3F8.^12^, ^13^

Although many adverse events (AEs) are common to all anti‐GD2 therapies, naxitamab requires a different approach to the management of AEs due to its shorter infusion duration and outpatient administration. It is therefore important to understand how these differences may impact care, including modifications that may be required to existing management strategies. In Trial 201, naxitamab AEs were mitigated with premedication and managed with timely recognition and intervention using protocol AE management guidelines and algorithms.

### 1.1 Clinical trial experience with naxitamab

**FIGURE 1:**
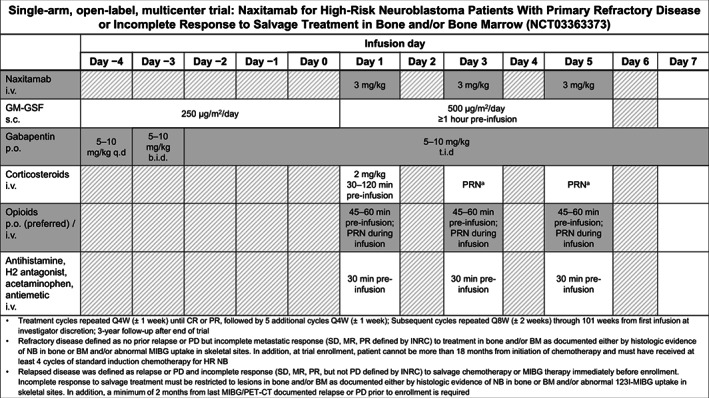
Trial 201: phase 2 trial treatment and supportive medication schedule. ^a^Stepped‐up dosing regimen for GM‐CSF based on Kushner et al. 1989.^12^ ^b^If grade 3 anaphylaxis or bronchospasm were experienced, repeat corticosteroid infusion for example, during infusions 2 and 3 within the same cycle, or infusion 1 of subsequent cycles. b.i.d., bis en die (twice a day); BM, bone marrow; CR, complete response; HR, high risk; INRC, International Neuroblastoma Response Criteria; i.v., intravenous; MIBG, 123I‐meta‐iodobenzylguanidine; MR, minor response; NB, neuroblastoma; p.o., oral; PD, progressive disease; PR, partial response; PRN, pro re nata (as needed); Q4W, every 4 weeks; Q8W, every 8 weeks; q.d. quaque die (once a day); s.c., subcutaneous; SD, stable disease; t.i.d, ter in die (three times a day)

Trial 201 is a phase 2, single‐arm, open‐label, international clinical trial designed to evaluate the efficacy and safety of naxitamab plus GM‐CSF in patients with HR neuroblastoma and primary refractory disease (refractory) or incomplete response to salvage therapy for relapsed or progressive disease (relapsed) limited to bone and/or BM (see Figure 1 for study definitions). Thirty‐six patients were evaluable for efficacy at the time of data cutoff of August 5, 2020: median age at enrollment was 5 years, 58% were male, 13% had *MYCN* amplification, and 88% had International Neuroblastoma Staging System (INSS) Stage 4 disease. Prior treatment included chemotherapy (100%), surgery (88%), radiation (38%), autologous stem cell transplantation (25%), and anti‐GD2 therapies (18%). Responses were assessed by independent pathology and imaging review committees using revised International Neuroblastoma Response Criteria (INRC).^14^ The overall response rate (ORR) was 58% (95% confidence interval [CI] 41–75%) with a 44% complete response (CR) rate.^8^

Forty‐eight patients who initiated naxitamab infusion were evaluable for safety. Per study protocol, all AEs were graded based on Common Terminology Criteria for Adverse Events (CTCAE) version 4.0.^15^ The most frequent AEs were hypotension (98%), pain (96%), urticaria (83%), pyrexia (79%), bronchospasm (67%), tachycardia (63%), cough (58%), vomiting (52%), and nausea (50%). Bronchospasm, hypotension, and hypertension were more frequent during the first treatment cycles, and the highest incidences of bronchospasm and hypotension were generally reported during the first infusion of each cycle. Serious AEs (SAE) were experienced by 19 patients (39%). Device‐related infections were the most common SAEs (five events), followed by hypotension and anaphylaxis (four events each), and pain and pyrexia (two events each). Naxitamab treatment was discontinued due to treatment‐related AEs in four patients (causal events: anaphylaxis, respiratory depression, urticaria, and hypotension).

Infusion‐rate interruptions or modifications due to AEs were experienced by 77% of patients. AEs generally occurred within 24 h of completing an infusion and often started as early as 3 min after initiating an infusion. For the majority of patients, their condition improved shortly after infusion completion. No patients experienced posterior reversible encephalopathy syndrome. No fatal AEs were reported.

### 1.2 Preparing for naxitamab infusion

#### 1.2.1 Staffing and education

Trial 201 staff were educated on the naxitamab safety profile and trained on protocol‐based AE management guidance. Investigators and supporting staff were familiarized with the practices of other sites to enhance the safe and effective use of naxitamab. It was important for the treating team (physicians and nurses) to be familiar with the anticipated AEs seen with naxitamab administration and to be able to respond rapidly and appropriately. Staff who were present by the bedside were therefore trained in resuscitation and treatment of severe hypotension.^16^

#### 1.2.2 Preparing patients and their caregivers for infusion

In view of the anticipated AEs (especially pain and hypotension), it is important that caregivers and patients (when age appropriate) are advised in advance as to what to expect during the naxitamab infusion. In cases where families have experience with dinutuximab or dinutuximab beta administration, it may be helpful to draw similarities (in terms of type of AEs) as well as to emphasize the differences (more acute timeframe of AEs with naxitamab and planned outpatient administration). Caregivers play a critical role in ensuring that patients are best prepared for naxitamab infusion, such as arriving for treatment on an empty stomach to minimize risk of nausea and vomiting; this is particularly important for the first infusion.

#### 1.2.3 Protocol‐recommended premedications and bedside medication

To reduce the frequency and severity of AEs, all patients received premedication before starting naxitamab infusion, both at home (when applicable) and at the treatment center. Table 1 summarizes the recommended premedications and bedside medications for mitigating and treating AEs observed with naxitamab. To adapt to the patient's specific needs and account for marked inter‐patient variation in some AEs (e.g., pain), modifications to supportive therapy and premedication doses may have been required after the first infusion. An individualized list of premedications should be made available for the specific patient for subsequent infusions. In addition, two intravenous (i.v.) accesses should be established prior to initiation of treatment to allow for infusion of both naxitamab and any supportive therapies as required. This can be either a double‐lumen central venous line or a port and peripheral venous line, and is based on patient age and body weight, as some patients who develop hypotension may require faster infusion rates for fluid resuscitation than can be met by the single‐lumen central venous access port that most patients have.

**TABLE 1:** Premedication and supportive therapy to manage adverse events associated with naxitamab therapy in Trial 201

Therapy	Indication	Dosage	Administration and timing
*At home*			
Gabapentin	Pain	5–10 mg/kg p.o. (max. 600 mg/day)	Day−4: 1×/day Day–3: 2×/day Day−2 and onwards until Day 7: 3x/day
*Premedication on day of infusion*			
Saline solution	Hypotension	Saline 10 mL/kg i.v. bolus	Over 1 h, just prior to naxitamab infusion
Opioid	Pain	Preferred: Oxycodone 0.1–0.2 mg/kg p.o. (max. 5 mg) or equivalent dose of oral opioids Alternative: Hydromorphone 3.75–15.0 μg/kg or morphine sulphate / morphine sodium chloride 0.025–0.1 mg/kg i.v. over 2–10 min	p.o.: 45–60 min before naxitamab i.v.: 15 min before naxitamab
Corticosteroid	Anaphylaxis	Methylprednisolone 2 mg/kg i.v. (max. 80 mg)	Mandated: Cycle 1 Day 1: 30 min–2 h before naxitamab PRN: After grade 3 anaphylaxis (or bronchospasm): at first infusion of a cycle: at first infusion in subsequent cycle After grade 3 anaphylaxis (or bronchospasm): at subsequent infusion At investigator's discretion when AEs were insufficiently controlled by other premedication
Antihistamine	Hypersensitivity	One of the following: Hydroxyzine 0.5–1.0 mg/kg p.o. or i.v. (max. 50 mg) Diphenhydramine 0.5–1.0 mg/kg i.v. (max. 50 mg) Cetirizine p.o. 2.5 mg if <20 kg; 5.0 mg if >20 kg; 10.0 mg if >12 years and > 30 kg Loratadine p.o. 5 mg if 2–5 years; 10 mg if >5 years Equivalent according to local SoC	30 min before naxitamab
Antipyretic	Pyrexia	Paracetamol (acetaminophen) 10–15 mg/kg p.o. or i.v. (max. 750 mg)	30 min before naxitamab
Antiemetic	Nausea, vomiting	Ondansetron 5 mg/m^2^ p.o. or i.v. or equivalent according to local SoC	30 min before naxitamab
H2 antagonist	Hypersensitivity, including urticaria	Local SoC	PRN before naxitamab
Anxiolytic	Anxiety	Lorazepam 0.01–0.02 mg/kg i.v. (max. 1 mg) or equivalent according to local SoC	PRN before naxitamab
*PRN (ready at bedside)*			
Opioid	Pain	Ready as precharged syringes: Hydromorphone 3.75–15.0 μg/kg i.v. over 1–5 min; or Morphine sulphate / morphine sodium chloride 0.025–0.1 mg/kg i.v. over 1–5 min	At the onset of pain Repeated every 5 min to reduce risk of hypotension for a total of four doses Additional doses given PRN at the discretion of the treating physician
NSAID	Pain	NSAID according to local SoC	
Cold/heat therapy	Pain	Cold and hot packs	Applied locally in any body area PRN
Anxiolytic	Anxiety	Lorazepam 0.01–0.02 mg/kg i.v. (max. 1 mg) or equivalent according to local SoC	
H1 receptor blocker	Bronchospasm/urticaria/anaphylaxis	Dexchlorpheniramine 0.15 mg/kg i.v. (max. 5 mg) or equivalent H1 receptor blocker according to local SoC	
Corticosteroid	Bronchospasm/anaphylaxis	Methylprednisolone 2 mg/kg (max. 80 mg) or equivalent corticosteroid	If glucocorticosteroid was not given as premedication If premedicated with glucocorticosteroid, dose reduction was considered at the investigator's discretion
Catecholamine	Hypotension/anaphylaxis	Epinephrine 0.01 mg/kg i.v./i.m (max. 0.5 mg)	
Bronchodilator	Bronchospasm	Salbutamol nebulized 2.5 mg if <20 kg; 5 mg if >20 kg; or Levalbuterol or comparable selective β2‐adrenergic receptor agonist (bronchodilator), according to local SoC	
Humidified oxygen	Hypoxia	Oxygen mask and inhalation equipment	
Racepinephrine	Stridor/bronchospasm	Racepinephrine or equivalent nebulized inhalant	
Naloxone	Respiratory depression	Naloxone i.v. 1 μg/kg/dose	Repeated every 2–3 min until response
Saline solution	Hypotension	Saline 10–20 ml/kg i.v. bolus over 5–15 min	

Abbreviations: AE, adverse event; i.m., intramuscular; i.v., intravenous; max., maximum; min, minute; NSAID, nonsteroidal anti‐inflammatory drug; p.o., oral; PRN, pro re nata (as needed); SoC, standard of care.

#### 1.2.4 Vital signs and frequency of monitoring

Vital signs, including heart rate, respiratory rate, body temperature, and blood pressure (BP), were monitored before, during, and after each infusion (Days 1, 3, and 5 of each cycle), and every hour during the postinfusion observation period (≥2 h after completion of treatment, or after last dose of any i.v. opioid). If any deviations were seen in vital sign values from those recorded preinfusion, the frequency of monitoring was increased. In addition, vital signs were taken before administering premedication and before pro re nata (as needed; PRN) pain management (e.g., with i.v. opioids or ketamine) during the naxitamab infusion. Additionally, peripheral oxygen saturation was measured when clinically indicated. Supplementary Table 1 provides an overview of vital sign assessment.

## 2 MANAGING AES WITH NAXITAMAB TREATMENT

The AEs observed with naxitamab are likely due to activation of the mAb target (GD2; e.g., pain and hypotension) and/or complement activation/deposition (e.g., fever and anaphylaxis).^17^ Some supportive therapies may aggravate specific AEs observed with naxitamab, such as opioid‐related exacerbation of hypotension. Patients should therefore be closely monitored during the infusion and the treating physician should always use best clinical judgment to ensure the safety of the patient.

The following sections summarize the main AEs associated with naxitamab, and how they were managed during Trial 201.

### 2.1 Pain

As GD2 is also present on the surface of neurons in peripheral nerve fibers, acute generalized pain during and following infusions is a known class effect of anti‐GD2 mAbs.^17^, ^18^, ^19^ The pain experienced with naxitamab infusion is often variable and may present as generalized, abdominal, extremity, bone, neck, back, noncardiac chest, flank, or musculoskeletal pain.^20^ In Trial 201, 96% of patients experienced pain, with grade 3 pain reported for 65% of patients.^8^ All patients received oral gabapentin at home, starting 5 days before the first naxitamab infusion, each day during Day1–5 of each cycle, and continued for 2 days postinfusion (12 days total). Oral (p.o.) or i.v. opioid premedication was administered at the treatment center before naxitamab infusion (Table 1); p.o. opioids were preferred as oral administration is associated with a reduced risk of AEs such as hypotension, respiratory suppression, and decreased responsiveness.^21^ If p.o. opioid therapy was not feasible, i.v. opioid was administered as premedication. The use of analgesics was adjusted based on the patient's pain experience and management regimen during the first naxitamab infusions.For breakthrough pain during the infusion, additional i.v. opioids were ready as precharged syringes and given at the onset of pain (Table 1). To reduce the risk of hypotension, low doses of opioids were selected to allow repeated administration every 5 min for up to a total of four doses, at the discretion of the treating physician. Naloxone was recommended to reverse potential opioid overdose resulting in hypotension, poor perfusion, and/or decreased consciousness.^22^ Nonpharmacological interventions, such as play therapy, distraction techniques (e.g., favorite toy, or watching favorite movie or show), and hot/cold packs, were also used for reducing therapy‐related pain.

While opioids are widely used to ameliorate pain during infusion of anti‐GD2 mAb therapies, they may not be tolerated or provide adequate pain relief for all patients, and so alternative approaches may be required.^23^ The pain associated with anti‐GD2 mAbs appears mediated in part through the N‐methyl‐D‐aspartate (NMDA) receptor, thus the use of an NMDA receptor antagonist, such as ketamine, may offer mitigation.^19^ Unlike opioids, ketamine is not typically associated with an increased risk of hypotension and may have mild hypertensive effect via catecholamine release^24^; this may be beneficial due to the elevated risk of hypotension seen with naxitamab use (see section on hypotension below).

Ketamine was permitted in Trial 201, in case of inadequate pain control or hypersensitivity to opioids. Ketamine, delivered as a 2 mg/kg i.v. bolus, was used by four patients at one of the Trial 201 sites, where it was shown to effectively control pain associated with naxitamab administration, providing an alternative for those patients in whom opioids were ineffective or contraindicated.^25^, ^26^ Both a bolus approach, administering ketamine pre and during infusion, and continuous low‐dose infusion have been used, reflecting site‐specific use of ketamine.If the patient experienced lingering pain following completion of the naxitamab infusion, acetaminophen, ibuprofen, and, if needed, p.o. opioids could be administered.

### 2.2 Hypotension

Management of hypotension presents unique challenges because of its complex pathophysiology: vasovagal reflexes or complement activation‐related pseudoallergy have been suggested,^27^, ^28^, ^29^ however impact on peripheral arterioles driven by GD2 activation of sympathetic nerves cannot be ruled out. Hypotension and unresponsiveness can also be caused or exacerbated by opioid‐induced histamine release.^30^ Patients must be carefully monitored, particularly when receiving opioids for breakthrough pain.^21^

In Trial 201, 98% of patients experienced hypotension, with grade 3–4 reported for 63% of patients.^8^ Investigators observed that hypotension during naxitamab infusion was generally associated with tachycardia and may be accompanied by a skin rash.

**FIGURE 2:**
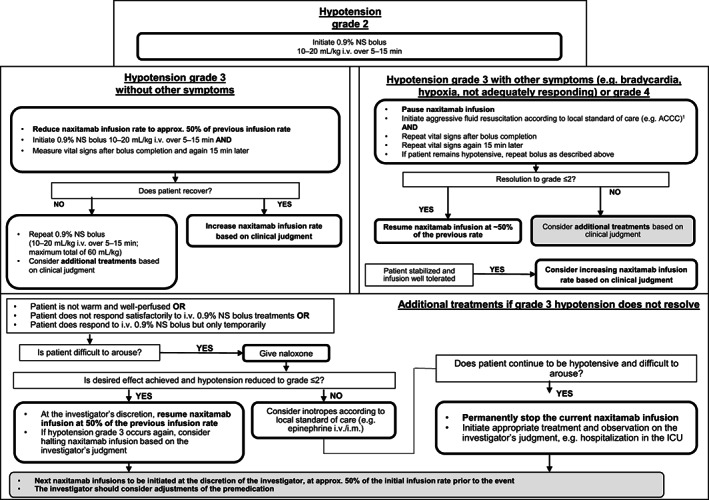
Algorithm for management of naxitamab infusion‐related hypotension. ^a^Based on CTCAE version 4.0. ACCC, American College of Critical Care Medicine; approx. approximately; CTCAE, Common Terminology Criteria for Adverse Events; ICU, intensive care unit; i.m., intramuscular; i.v., intravenous; min, minute; NS, normal saline

Hypotension was managed with saline boluses during the infusion; an i.v. saline infusion was given during the first 15 min, and additional fluids were prepared and connected to the patient's i.v. line prior to initiating naxitamab infusion, to be given if needed. Naxitamab infusion rate reduction or interruption was determined by the severity of AE (Figure 2). It is recommended that patients receiving naxitamab are confined to bed for the duration of the administration and not permitted to stand or walk to the bathroom in view of the risk of orthostatic hypotension; where possible, the bed should be full size to allow parents to lie with the child, and should allow for Trendelenburg position (e.g., head declined below feet). If hypotension is refractory and developed following opioid administration, the use of naloxone may be required.

In rare cases, intramuscular or i.v. epinephrine was required if patients did not respond sufficiently to fluid resuscitation (Table 1). Current guidance, implemented during the clinical trial conduct, recommends i.v. administration of normal saline 10 mg/kg over 1 h just before the start of each naxitamab infusion. Due to the data‐cut reported and timing of the amendment, none of the patients in the trial data discussed here received preinfusion hydration.

### 2.3 Bronchospasm and hypoxia

Bronchospasm and other respiratory AEs occurred as a reaction to the mAb infusion or were triggered by pain (e.g., breath‐holding leading to hypoxia) during the infusion.

During Trial 201, bronchospasm was experienced by 67% (any grade) and 21% (grade 3–4), and grade 3–4 hypoxia was reported for 10% of patients.^8^

**FIGURE 3:**
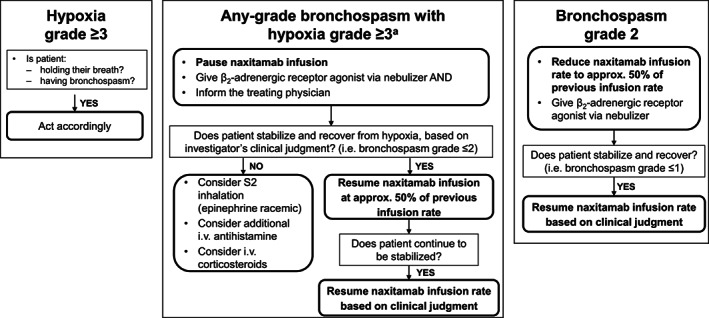
Algorithm for management of naxitamab‐related bronchospasm and hypoxia. ^a^Based on CTCAE version 4.0. approx., approximately; CTCAE, Common Terminology Criteria for Adverse Events; i.v., intravenous

Supportive medication to counteract respiratory problems was administered PRN and kept ready at bedside during naxitamab administration (Table 1). As detailed below, the Trial 201 protocol was adapted to mandate corticosteroid premedication before the first infusion of Cycle 1. Humidified oxygen inhalation equipment was used if hypoxia occurred. Infusion interruption or rate reduction was determined by the severity of the AE (Figure 3).

### 2.4 Anaphylaxis and other hypersensitivity reactions

Infusions of mAbs can give rise to hypersensitivity reactions including anaphylaxis.^28^ Patients were therefore carefully monitored for any signs or symptoms occurring during or after an infusion that could indicate serious hypersensitivity or anaphylaxis, such as facial swelling, urticaria, or difficulty breathing. Patients and their caregivers were also educated regarding these signs and advised to immediately report their occurrence.

During Trial 201, patients were withdrawn from naxitamab treatment if they experienced grade 3 anaphylaxis that was unresponsive to medical intervention, or grade 4 anaphylaxis. A total of 4 SAEs of anaphylaxis were reported for 3 patients (6%); 2 patients (8%) permanently discontinued naxitamab due to anaphylaxis. All 3 patients recovered from the anaphylaxis event.^8^

Corticosteroid premedication is now recommended for all patients, 30–120 min prior to the first infusion of naxitamab to mitigate the risk of anaphylactic reaction and bronchospasm.^31^ The mandated steroid premedication before the first dose of naxitamab was implemented during the clinical trial conduct; only 25 patients in the safety population (n = 48) received corticosteroid premedication. Only 1 corticosteroid dose was mandated, for example, before the first naxitamab dose (Cycle 1 Day 1), however this premedication could also be given before a subsequent infusion if a grade 3 anaphylaxis or bronchospasm occurred during the previous infusion, or before the first infusion of a given cycle if an event occurred during the first infusion of the previous cycle (Table 1).

### 2.5 Urticaria

The presentation of urticaria during and after the infusion, was highly variable (focal as well as widespread) and previous patterns of urticaria did not necessarily predict future presentations. During Trial 201, 83% of patients experienced urticaria, with grade 3 reported for 29% of patients.^8^

To minimize urticaria and other skin‐related hypersensitivity reactions, an i.v. or p.o. antihistamine was given 30 min before infusion. H2 blockers such as famotidine were also used as a premedication to mitigate urticaria.

### 2.6 Hypertension

During Trial 201, 35% of patients experienced hypertension, all under grade 3.^8^ Most hypertension events occurred on the day of naxitamab infusion, but hypertension was reported up to 9 days postinfusion.

Appropriate management of hypertension was based on BP percentile according to age, sex, and weight and local standards of care.^32^ In order to be prepared for any BP deviation, the maximum BP was calculated preinfusion and clearly marked on the patient's chart to allow rapid identification of hyper‐ or hypotension.

**FIGURE 4:**
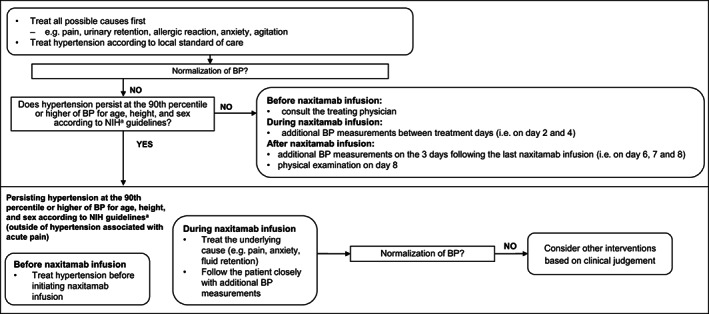
Algorithm for management of naxitamab‐related hypertension, outside of hypertension associated with acute pain. BP, blood pressure; NIH, National Institute for Health. ^a^Blood pressure levels for boys and girls by age and height percentile. National Heart, Lung and Blood Institute^32^

Figure 4 shows the protocol‐based treatment algorithm for management of hypertension, with Supplementary Figure 1 illustrating the dose‐modification algorithm for hypertension.

BP was monitored closely during the infusion, and daily during the treatment cycle (e.g., Days 1–5 of each cycle) until 3 days after the last infusion (e.g., Days 6–8) if the BP remained >90%. This is important as two events of posterior reversible encephalopathy syndrome were observed in the 12–230 trial, seemingly linked to hypertension associated with naxitamab, however there have been no events reported in Trial 201. Mitigation efforts for hypertension introduced during Trial 201 may have had an impact.

Patients with hypertension postinfusion may have received antihypertensive medication(s) and, if needed, were to be admitted to an inpatient service for monitoring if their BP remained >99%. If patients were receiving antihypertensive medications on the day of infusion, withholding this treatment until after the infusion was considered, to avoid exacerbation of hypotension during the infusion; otherwise naxitamab should be discontinued in patients with persistent hypertension that did not respond to antihypertensive interventions on the day of infusion.

### 2.7 Other AEs

Similar to other mAb therapies, fever and gastrointestinal (GI) AEs may be present but can be minimized with appropriate premedication and patient education (Table 1).

Vomiting and diarrhea were experienced by 52% (2% grade 3) and 46% (4% grade 3) of patients, respectively.^8^ GI AEs could be due to medications such as ketamine or opioids, vaso‐vagal reflexes, the patient having a large breakfast prior to the infusion, or pain‐related agitation. To minimize the risk of GI discomfort, at some centers, patients were advised to minimize oral intake on the morning prior to naxitamab infusion. Premedication with an antiemetic 30 min before naxitamab infusion (e.g., ondansetron or equivalent) was also used to minimize GI‐related AEs.

Pyrexia was experienced by 79% of patients, with 1 patient experiencing grade 3 pyrexia classed as an SAE.^8^ To reduce the risk of fever, patients were premedicated with an antipyretic, such as acetaminophen, 30 min before naxitamab infusion.

### 2.8 Neurotoxicity

In addition to pain, other neurologic conditions have been observed with naxitamab infusions, such as peripheral neuropathy and neurologic disorders of the eye. These neurologic disorders seen with naxitamab in Trial 201 were grade 1–2, with 8% experiencing sensory peripheral neuropathy and 31% experiencing some form of eye disorder.

## 3 POSTNAXITAMAB INFUSION

Patients were observed for a period of at least 2 h following completion of naxitamab infusion or the last dose of i.v. opioid and could then be discharged provided AEs had resolved and vital signs were acceptable.

## 4 DISCUSSION AND CONCLUSION

In Trial 201, of the 48 patients with available safety data, ≥50% of patients experienced hypotension, pain, urticaria, pyrexia, bronchospasm, tachycardia, cough, vomiting, and nausea; naxitamab‐related SAEs, including hypotension and anaphylactic reaction, were reported for 11 patients. These AEs seen with naxitamab were manageable with appropriate and timely intervention, including premedication before infusion, close monitoring and supportive therapies during infusions, and observation postinfusion. It is critical that teams administering naxitamab are aware of likely AEs and ready to manage these promptly. Infusion rate reductions or interruptions were employed, based on the severity of the AEs. Discontinuations were minimized via appropriate management of AEs, allowing patients to receive full treatment and reducing the risk of hospitalization or delays to discharge. The successful management of AEs during naxitamab infusion was evidenced in Trial 201 where 96% of infusions were administered in the outpatient setting and 99% of infusions were administered at the full naxitamab dose.^9^

AEs with a complex pathophysiology, such as hypotension, hypoxia, and bronchospasm, which can be influenced by supportive therapies and the patient's general condition, can be complicated to designate, report, and manage. While some AEs reported can be classified as infusion‐related reactions, taking a specific approach, if clinically feasible, for each AE, such as hypotension and bronchospasm, facilitates proper management and avoids misclassification. For example, hypotension and bronchospasm can resemble anaphylaxis, and should be distinguished from such to avoid unjustified discontinuing naxitamab. Algorithms were established to manage the most frequent and most severe AEs.

Reflecting the experience to date, the Trial 201 protocol now includes guidelines/recommendation for mitigation of hypotension via saline pretreatment (10 ml/kg over 1 h; before initiating naxitamab infusion). This aligns with guidance for other anti‐GD2 mAbs, such as dinutuximab, for which hydration prior to infusion is recommended.^10^

The treatment team may already be familiar with anti‐GD2 mAbs for neuroblastoma from their experience with dinutuximab and/or dinutuximab beta. However, the outpatient administration and shorter infusion time with naxitamab necessitates an adapted approach to management as well as additional education and training for clinical team members.

As with administration of any anti‐GD2 mAbs, it is essential to prepare patients and their caregivers for naxitamab treatment. Ensuring the patients, and crucially the caregivers, understand what to expect during treatment helps to optimize naxitamab infusion experience, enhance patient care, and reduce the burden on patients, caregivers, and healthcare providers. Discussing the supportive treatment approach and patient preference before initiation of treatment is crucial for AEs such as pain, for which physicians should take into consideration inter‐patient variation in both pain experience and analgesia needs.

The Trial 201 premedication, supportive medications, and AE management guidelines are based on established protocol algorithms and supplemented with input from investigators and data monitoring committees, as well as experience from compassionate use. The recommendations and algorithms presented here may help clinicians use naxitamab safely and effectively.

## AUTHOR CONTRIBUTIONS

**Jaume Mora:** Conceptualization (equal); investigation (equal); methodology (equal); supervision (equal); writing – original draft (equal); writing – review and editing (equal). **Godfrey C. Chan:** Conceptualization (equal); investigation (equal); methodology (equal); writing – original draft (equal); writing – review and editing (equal). **Daniel A. Morgenstern:** Conceptualization (equal); investigation (equal); methodology (equal); writing – original draft (equal); writing – review and editing (equal). **Karsten Nysom:** Conceptualization (equal); investigation (equal); methodology (equal); writing – original draft (equal); writing – review and editing (equal). **Melissa K. Bear:** Conceptualization (equal); investigation (equal); methodology (equal); writing – original draft (equal); writing – review and editing (equal). **Karen Tornøe:** Conceptualization (equal); data curation (equal); methodology (equal); writing – original draft (equal); writing – review and editing (equal). **Brian H. Kushner:** Conceptualization (equal); investigation (equal); methodology (equal); supervision (equal); writing – original draft (equal); writing – review and editing (equal).

## CONFLICT OF INTEREST

Mora J: Consulting fees from Y‐mAbs Therapeutics. Chan GCF: No relevant disclosures. Morgenstern DA: Advisory boards/consultancy for Boehringer‐Ingelheim, Clarity Pharmaceuticals, EUSA Pharma, Roche, and Y‐mAbs Therapeutics. Nysom K: Advisory board for Bayer AG, EUSA Pharma, and Y‐mAbs Therapeutics; teaching for Bayer AG; and consultancy for Y‐mAbs Therapeutics. Bear MK: No relevant disclosures. Tornøe K: Employed by and equity with Y‐mAbs Therapeutics. Kushner BH: No relevant disclosures.

## Supporting information


**Appendix S1** Supporting information

## Data Availability

Data sharing is not applicable to this article as no new data were created or analyzed in this study.
