# Supplementary material for: Outpatient administration of naxitamab in combination with granulocyte‐macrophage colony‐stimulating factor in patients with refractory and/or relapsed high‐risk neuroblastoma: Management of adverse events
Source: Cancer Rep (Hoboken). 2022 May 17;6(1):e1627. doi: 10.1002/cnr2.1627 (PMC9875606; doi:10.1002/cnr2.1627)
Supplement: Supplementary file 1 — Appendix S1 Supporting information [file CNR2-6-e1627-s001.docx]

# SUPPLEMENTAL INFORMATION

## **SUPPLEMENTAL TABLE 1 Schedule of vital sign assessments during screening and on day of infusion (Days 1, 3, and 5 of each cycle)**

| **Schedule** | **Screening (Day −25 to −4)** | **Before infusion** | | **During infusion** | | **After infusion** | | **Hypoxia** |
| --- | --- | --- | --- | --- | --- | --- | --- | --- |
|  |  | **Before premedication** | **Before naxitamab infusion** | **During naxitamab infusion** | **Before PRN opioids/ analgesia** | **Immediately post- infusion** | **During  2-hour post-infusion observation** | **Before, during, and after an event** |
| Vital Sign | | | | | | | | |
| Blood pressure | ✓ | ✓ | ✓ | ✓ | ✓ | ✓ | ✓ | 🗶 |
| Heart rate | ✓ | ✓ | ✓ | ✓ | ✓ | ✓ | ✓ | 🗶 |
| Respiratory rate | ✓ | ✓ | ✓ | ✓ | ✓ | ✓ | ✓ | 🗶 |
| Body temperature | ✓ | ✓ | ✓ | ✓ | ✓ | ✓ | ✓ | 🗶 |
| Peripheral oxygen saturation^a^ | ✓ | 🗶 | 🗶 | 🗶 | 🗶 | 🗶 | 🗶 | ✓ |

^a^Peripheral oxygen saturation is constantly monitored during an infusion; values to be recorded where indicated e.g. at screening and in the event of hypoxia.

Abbreviations: PRN, pro re nata (as needed).

## SUPPLEMENTARY FIGURE 1 Dosage modification algorithm for hypertension


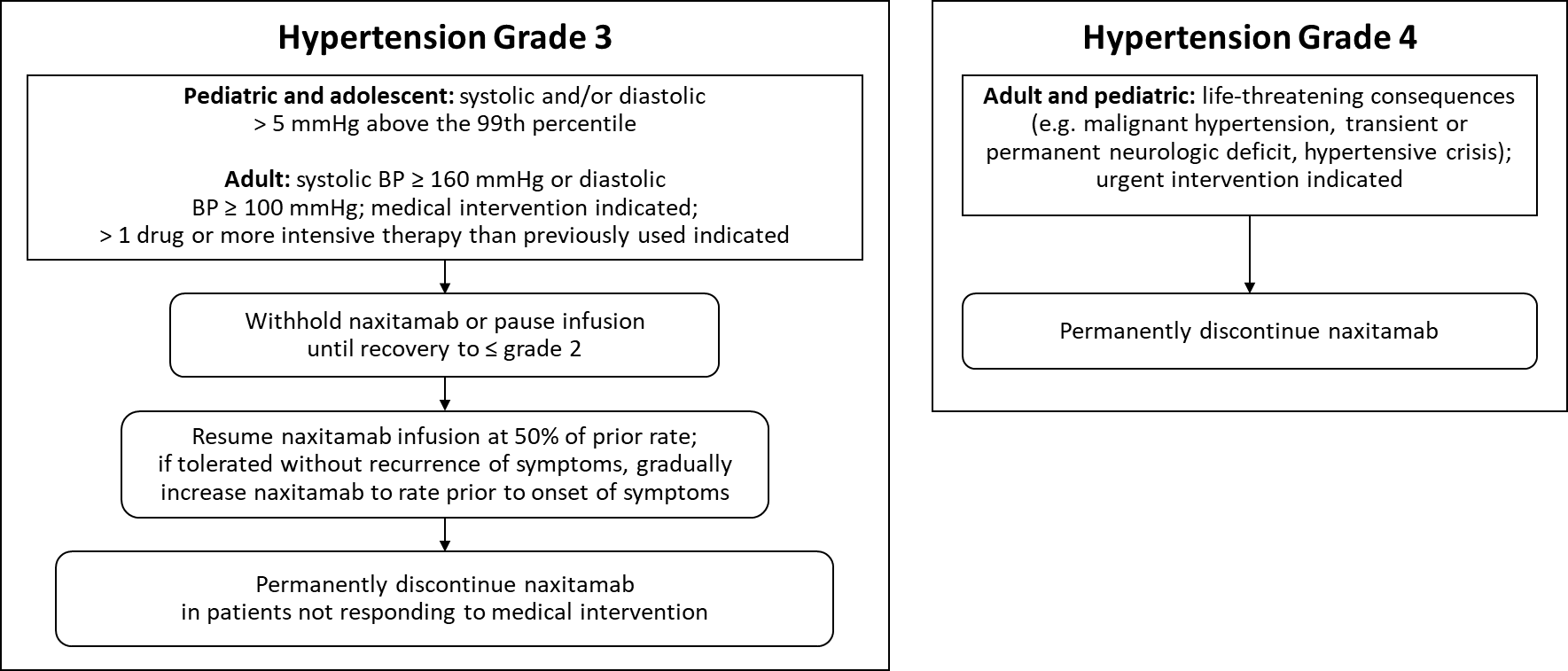


**Abbreviations**: BP, blood pressure.
